# Serum Concentrations of Ubiquitin C-Terminal Hydrolase-L1 and Glial Fibrillary Acidic Protein after Pediatric Traumatic Brain Injury

**DOI:** 10.1038/srep28203

**Published:** 2016-06-20

**Authors:** Stefania Mondello, Firas Kobeissy, Annarita Vestri, Ronald L. Hayes, Patrick M. Kochanek, Rachel P. Berger

**Affiliations:** 1 Department of Biomedical and Dental Sciences and Morphofunctional Imaging, University of Messina, Messina, Italy; 2 Department of Psychiatry, University of Florida, Gainesville, FL, USA; 3 Department of Public Health and Infectious Disease, Sapienza University of Rome, Rome, Italy; 4 Banyan Biomarkers, Inc. Alachua, FL, USA; 5 Department of Critical Care Medicine, Safar Center for Resuscitation Research, Pittsburgh, PA, USA; 6 Children’s Hospital of Pittsburgh of UPMC, Pittsburgh, PA, USA

## Abstract

Objective reliable markers to assess traumatic brain injury (TBI) and predict outcome soon after injury are a highly needed tool for optimizing management of pediatric TBI. We assessed serum concentrations of Glial Fibrillary Acidic Protein (GFAP) and Ubiquitin C-Terminal Hydrolase-L1 (UCH-L1) in a cohort of 45 children with clinical diagnosis of TBI (Glasgow Coma Scale [GCS] 3–15) and 40 healthy subjects, evaluated their associations with clinical characteristics and outcomes, and compared their performance to previously published data on two well-studied blood biomarkers, S100B and MBP. We observed higher serum levels of GFAP and UCH-L1 in brain-injured children compared with controls and also demonstrated a step-wise increase of biomarker concentrations over the continuum of severity from mild to severe TBI. Furthermore, while we found that only the neuronal biomarker UCH-L1 holds potential to detect acute intracranial lesions as assessed by computed tomography (CT), both markers were substantially increased in TBI patients even with a normal CT suggesting the presence of undetected microstructural injuries. Serum UCH-L1 and GFAP concentrations also strongly predicted poor outcome and performed better than S100B and MBP. Our results point to a role of GFAP and UCH-L1 as candidate biomarkers for pediatric TBI. Further studies are warranted.

Traumatic brain injury (TBI) is a leading cause of death and acquired disability among children and adolescents world-wide^1^. After TBI occurs, children can either demonstrate enhanced recovery rates related to plasticity or experience prolonged symptoms and poorer outcome compared to adults^2^,^3^. Despite growing awareness of the risk of long-term morbidity and better understanding of biological mechanisms underpinning the enhanced vulnerability of the developing brain^3^,^4^, pediatric TBI remains a diagnostic, prognostic and therapeutic challenge to the clinician. Thus, circulating biomarkers of brain damage that can reliably detect injury to the central nervous system (CNS) and/or capture the underlying physiological and molecular processes and the potential for recovery would be valuable tools to complement currently available clinical data and aid in medical decision-making^5^.

Over the past few years, there have been a large number of studies focusing on pathobiologically and structurally diverse biomarkers such as S100B and myelin-basic protein (MBP) which can be measured in blood. Overall, these studies have demonstrated statistically higher concentrations of these biomarkers in children with TBI compared with controls, but these differences have not translated into clinical utility^6^,^7^,^8^. This may be attributable to the limitations of the biomarkers selected including extra-cranial sources, high normative concentrations in young children, and delayed appearance of the biomarker in serum after injury^9^,^10^,^11^. As a result, researchers have undertaken great efforts in identifying novel/complementary blood-based biomarkers of injury, which might help to overcome these limitations.

Ubiquitin C-terminal hydrolase (UCH-L1) is a proteolytically stable and abundant protein found almost exclusively in the cytoplasm of neurons. Previous adult studies have demonstrated that it is increased in serum after TBI^12^,^13^,^14^. To date, a single exploratory study by our group evaluated UCH-L1 concentrations in pediatric TBI and demonstrated that increased serum concentrations correlated with outcome^15^. Glial fibrillary acidic protein (GFAP), an astrocyte-specific cytoskeleton protein, is a well-established marker of glial damage in several traumatic neurological disorders^16^. Numerous adult studies have reported significantly raised serum concentrations of GFAP after TBI and their correlations with injury severity and outcome^13^,^17^,^18^. Increased serum GFAP has also been reported in children with severe TBI and concussions^19^,^20^. These findings indicate that UCH-L1 and GFAP may therefore be used to identify brain damage and assess the magnitude of injury in head-injured children.

In this study, we examined whether or not serum concentrations of UCH-L1 and GFAP were significantly elevated in children who suffered mild to severe TBI compared with uninjured controls, and determined their relationship with clinical characteristics and outcomes. We also compared their performance to two-well studied biomarkers - S100B and MBP - using previously published data from our group^21^,^22^.

## Results

### Demographics

The study included 45 cases and 40 controls. The mean (SD) age of cases was 3.8 (3.7) years; 62% were male. Among controls, the mean (SD) age was 3.9 (3.8) years, 58% were male. Cases and controls were well-matched with regard to demographic characteristics. The median (range) Glasgow Coma Scale (GCS) score was 10 (3–15). Of the cases, 19 (42%) had severe TBI, 6 (13%) had moderate TBI and 20 (45%) had mild TBI. Table 1 displays the demographic and clinical characteristics of the study population stratified according to the GCS score. Among the 45 cases, 10 (22%) had a negative CT and 6 (13%) an isolated skull fracture without evidence of intracranial injury (ICI). The median of GOS scores at 6 months post-injury was 5. Twenty-nine subjects had a favorable 6-month outcome (GOS 4–5) (Table 1).

### Serum Concentrations of GFAP, UCH-L1, S100B and MBP

Median (range) time between injury and biomarker measurement was 4.7 (0.5–20.6) hours after injury. Serum GFAP and UCH-L1 were significantly higher in cases vs. controls. S100B was also increased in TBI patients compared with controls; while there was no difference in serum MBP. Table 2 displays the median (interquartile range) serum concentrations of TBI biomarkers in the study populations.

Among controls, both UCH-L1 and S100B concentrations correlated with age (*R* = *−0.45*, *p* = 0.003 and *R* =* −0.55*, *p* = 0.0003, respectively). In particular, UCH-L1 concentrations during the first year of age were significantly higher than those measured at later ages (0.16 vs. 0.07 ng/ml, *p* = 0.003), with the highest individual concentrations observed in infants <3 months of age. No other correlations were found. Among cases, there was a weak negative correlation between UCH-L1 and age (*R* = −0.38, *p* = 0.01), though the correlation was no longer significant after adjusting for injury severity. Biomarker concentrations were not related to gender and there was no correlation with the time after injury when blood was collected. Among cases, GFAP and UCH-L1 concentrations were strongly correlated (*R* = *0.61*, *p* < 0.0001) as was GFAP and S100B (*R* = 0.56, *p* = 0.0002). S100B concentrations also correlated with UCH-L1 (*R* = *0.73*, *p* < 0.0001).

The diagnostic accuracy of both GFAP and UCH-L1 for differentiating cases and controls was good (AUCs 0.89 [95% CI 0.82 to 0.96] and 0.86 [95% CI 0.78 to 094], respectively). The sensitivity of GFAP and UCH-L1 was high (89% and 100%, respectively), although the specificity was moderate to low (63% and 20%, respectively). Diagnostic accuracy was significantly higher for GFAP compared to S100B (AUC 0.79 [95% CI 0.69 to 0.90] or MBP (AUC 0.44 [95% CI 0.31 to 0.57]) (*p* = 0.035 or *p* < 0.0001) and for UCH-L1 compared to MBP (*p* < 0.0001). Addition of S100 and MBP did not improve AUC values of GFAP and UCH-L1.

### Serum Biomarker Concentrations in Relation to TBI Severity and Imaging

**Figure 1:**
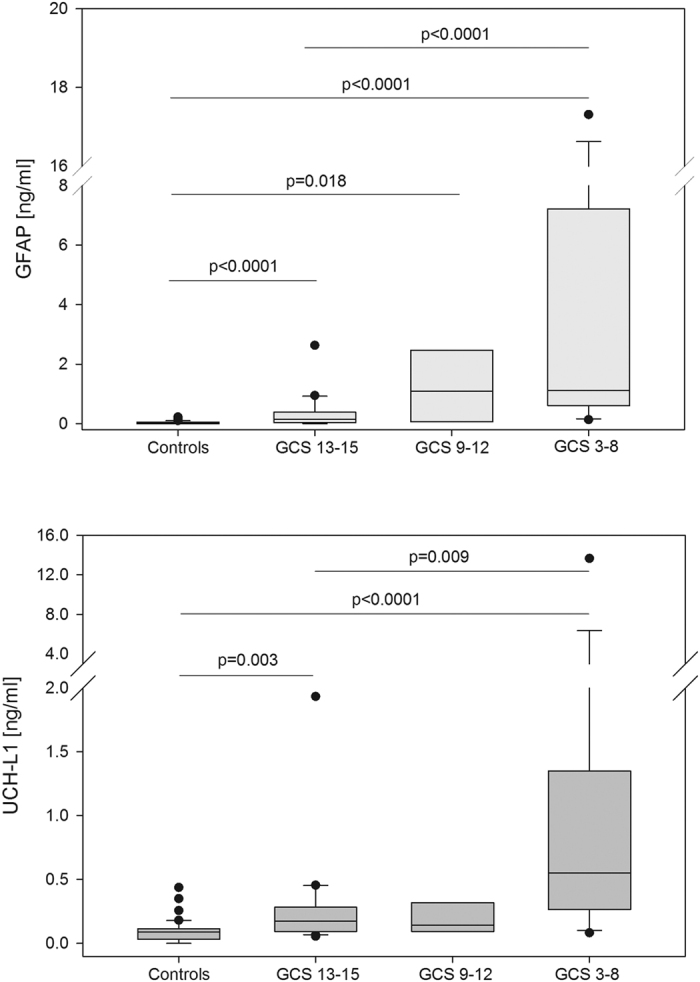
Box-and-whisker plots demonstrating serum GFAP and UCH-L1 concentrations cases with mild (GCS 13 to 15), moderate (GCS 9 to 12) or severe TBI (GCS 3 to 8) compared with controls. The black horizontal line in each box represents the median, with the boxes representing the interquartile range. Significant differences are indicated (Jonckheere-Terpstra test).

Serum GFAP, UCH-L1 and S100B concentrations were negatively correlated with the GCS score on admission (GFAP, R = −0.59, *p* < 0.0001; UCH-L1, R = −0.53, *p* = 0.0002; S100B, R = −0.47, *p* = 0.002). A highly statistically significant trend for increasing concentration of GFAP and UCH-L1 across severity groups/categories was found (*p* < 0.0001, and *p* < 0.0001, respectively, Jonckheere-Terpstra test) (Fig. 1). In particular, patients with severe TBI had significantly higher GFAP and UCH-L1 concentrations than those with mild TBI (median GFAP, 1.12 vs 0.15 ng/ml, *p* < 0.0001; median UCH-L1, 0.55 vs 0.18 ng/ml, *p* = 0.009) (Fig. 1). Further, both GFAP and UCH-L1 levels were significantly higher in patients with mild TBI compared with controls. More specifically, GFAP and UCH-L1 were able to differentiate patients with mild TBI from controls, with an AUC of 0.81 (95% confidence interval [CI] 0.68 to 0.94) and 0.81 (95% CI 0.70 to 0.92), respectively.

**Figure 2:**
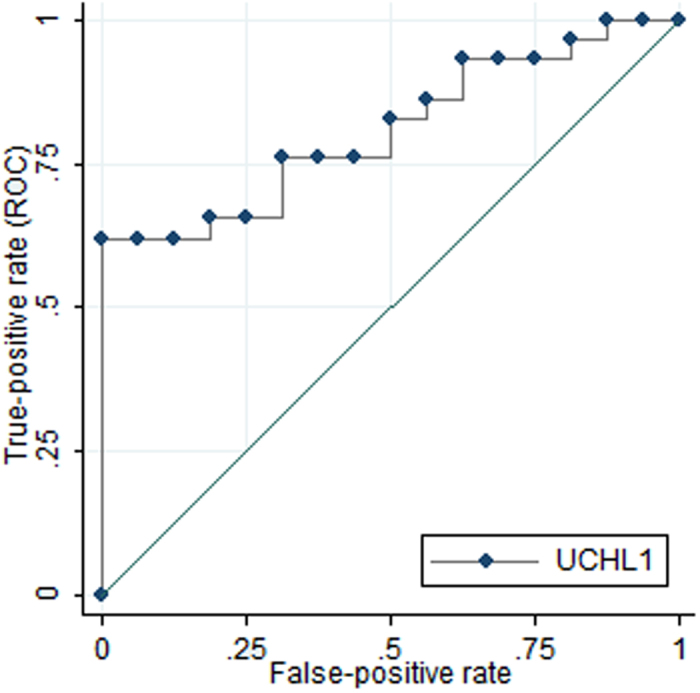
Receiver operating characteristic curves demonstrating the diagnostic accuracy of serum UCH-L1 concentrations for discriminating between patients who have relevant intracranial lesions on CT scans and those with a normal CT.

Among cases, UCH-L1 concentrations were significantly higher in patients with ICI compared with those with both a negative CT (*p* = 0.004) or skull fracture (*p* = 0.02). Conversely, GFAP concentrations did not differ between these groups, though there was a trend towards higher GFAP concentration in children with positive vs. negative CT. Children with a negative CT or skull fracture had increased levels of both UCH-L1 and GFAP compared with controls (Table 3). Using ROC curve analysis, a UCH-L1 cut-off point of 0.09 ng/ml was derived yielding a sensitivity of 93% and a specificity of 25% for the detection of ICI (AUC 0.81 [95% CI 0.68 to 0.93], *p* = 0.0008) (Fig. 2). GFAP was not able to discriminate between cases with and without ICI (AUC 0.59, *p* = 0.31). Inclusion of GFAP, S100 and MBP did not improve upon UCH-L1 alone in discriminating between patients who have intracranial lesions detectable on CTs and those with normal CTs.

### Serum Biomarker Concentrations in Relation to Outcome

The concentration on admission of GFAP, UCH-L1 and S100B correlated with the GOS score (GFAP, R = −0.46, *p* = 0.003; UCH-L1, R = −0.61, *p* < 0.0001; S100B, R = −0.44, *p* = 0.009), but not with MBP. Serum GFAP, UCH-L1 and S100B levels were significantly higher in children with unfavorable outcome than in patients with favorable outcome (median GFAP, 1.12 vs 0.27 ng/ml, *p* = 0.013; median UCH-L1, 0.92 vs 0.18 ng/ml, *p* = 0.0005; median S100B, 0.06 vs 0.03 ng/ml, *p* = 0.007), but MBP concentrations did not differ between these subpopulations. The diagnostic accuracy of serum GFAP and UCH-L1 for the prediction of unfavorable outcome were 0.76 (95% CI 0.60 to 0.92) and 0.86 (95% CI 0.72 to 1.00), respectively. A cut-off of 16.97 ng/ml for GFAP and 2.22 ng/ml for UCH-L1 yielded a diagnostic specificity of 100%, while sensitivities were 9% and 27%, respectively. The combination of the 2 markers did not provide a higher level of predictive power compared to UCH-L1 alone.

## Discussion

This exploratory study evaluated glial and neuronal proteins in 45 children representing the entire clinical TBI spectrum (GCS 3–15) and demonstrated significantly increased serum GFAP and UCH-L1 concentrations after injury. Importantly, we demonstrated a step-wise increase of biomarker concentrations over the continuum of severity from mild to severe TBI providing evidence that concentrations of GFAP and UCH-L1 reflect the magnitude of brain injury. Furthermore, our results indicate that UCH-L1 holds potential to detect acute intracranial lesions as assessed by CT and both markers might be used to predict outcome in pediatric TBI.

An intriguing observation arising from our study is that the concentrations of GFAP and UCH-L1 were highest in TBI subjects with ICI, but also increased in patients with a skull fracture and in those with a negative CT. This suggests the possibility of undetected brain damage in these subjects. Consistently with this hypothesis, a growing number of studies have demonstrated that CT provides poor sensitivity compared to advanced MRI methods for detecting, quantifying and characterizing small structural lesions and pathophysiologic alterations that can occur following TBI^23^,^24^,^25^. In addition, our group, along with others, has shown increased serum biomarker concentrations in CT-negative TBI patients with either persistent clinical symptoms or subtle intracranial abnormalities on highly sensitive imaging techniques^26^,^27^,^28^,^29^. Thus, it is plausible that the observed biomarker release in the cases with skull fracture or a negative CT in our study may have resulted from a limited structural damage, molecular perturbation, or specific patho-anatomic types of TBI, such as diffuse axonal injury or microbleeds, that were overlooked or remained undetected by CT^30^,^31^,^32^. As such, both GFAP and UCH-L1 may be relevant diagnostic adjuncts and possibly guide and optimize the use of advanced diagnostic imaging in pediatric TBI. Furthermore, these observations suggest caution in using CT as a gold standard to determine the presence of brain injury and judge the performance of circulating biomarkers, as they presumably explain the poor specificity of UCH-L1 for ICI as assessed by CT. Yet, the conclusions that can be drawn from our exploratory study are limited; a rigorous validation of these markers in combination with high-resolution MRI and other novel imaging modalities is needed.

In this study, we found that serum GFAP and UCH-L1 within 24hrs after injury correlate with GCS, with levels substantially raised in patients more severely injured (GCS 3–8) compared with those with mild TBI (GCS 13–15). Of note, some variability in biomarker levels, especially UCH-L1, was observed in children wih GCS between 9 and 12, which might be due to the small number of subjects in this group (n = 6). An alternative explanation is that this reflects some inconsistencies between biomarker and GCS assessment, as a consequence of the limited ability of the GCS score to stratify children with TBI, particularly in the grey zone of neurotrauma^33^,^34^. Supporting this interpretation is the low incidence of ICI and unfavorable outcome in children with moderate TBI as compared with those with mild TBI (Table 1).

Furthermore, our results are in line with previous studies showing that UCH-L1 and GFAP within the first 24hrs of admission predicted long-term clinical outcome^15^,^35^. Importantly both UCH-L1 and GFAP demonstrated 100% of specificity for the identification of poor outcome in our sample, though the sensitivity was low (27% and 9%, respectively). We speculate that the false negative misclassifications may have resulted from the too broad sampling time window (24 hours). The low sensitivity may also be explained by the fact that later secondary brain insults and other complications, which could not be captured by the initial biomarker levels, potentially contributed to poor outcome. However, there seems to be the potential for a further increase in the prognostic accuracy of UCH-L1 and GFAP by including other relevant clinical variables and defining optimal blood sampling time windows for the estimation of biomarkers.

Taken together, our findings provide compelling evidence that these biomarkers may be useful in risk stratification and support the idea that the use of a biomarker-based classification of injury severity, possibly in combination with clinical and imaging data, may improve categorization and identification of subsets of children at risk for poor outcome across the spectrum of TBI severity. These observations have high clinical relevance as a classification system for pediatric TBI using acute serum markers may not only be useful in diagnosis and prognosis but could also provide important insights into the injury-specific and patient-specific vulnerability facilitating early clinical interventions or stratification into clinical trials^36^.

Emerging technologies providing sensitivity and precision that are increased an order of magnitude over the present assays are now becoming available^37^,^38^ and are likely to offer the possibility of fully characterizing the distribution of biomarker in healthy individuals as well as detecting very small changes in biomarker concentrations in patients with TBI. In particular, in the present study using technically advanced GFAP and UCH-L1 assays, we were able to detect low biomarker concentrations in healthy children. In those subjects, a significant correlation between serum UCH-L1 and age was found. Although, serum UCH-L1 concentrations have been reported to increase with age in young healthy adults^39^, to our knowledge, the relationship in childhood and adolescence has not yet established. Our observation of age-related changes in serum UCH-L1 in children is intriguing. Potential explanations for this phenomenon are the underdevelopment and the greater permeability of the blood–brain barrier (BBB) in infants^40^ as well as unique age-related differences in brain biology and ongoing CNS developmental processes occurring during early stages of life. To this end, besides the demonstrated role of UCH-L1 on neuronal function and survival, recent studies also support its participation in regulation of neurogenesis and differentiation of neural progenitor cells^41^.

It is also noteworthy that, although GFAP and S100B are both glial markers, our results support the idea that GFAP can be a better biomarker of TBI than S100B. This may be attributable to two of the known limitations of S100B – its age dependence, particularly in very young children^42^,^43^, and its lack of specificity in patients with extracranial injuries^44^,^45^. To this end, future studies including children with acute orthopedic injuries without CNS involvement would be extremely valuable, as they also would allow to confirm the specificity of GFAP and UCH-L1 in pediatric TBI diagnostics.

There are several limitations of this study. First, our study has a relatively modest sample size that precluded meaningful multivariate analysis for independent predictors of outcomes. Second, because subjects were not enrolled consecutively, but rather when study personnel were available to obtain consent, the proportion of cases with severe TBI and those with a positive CT is artificially increased. Third, the heterogeneity of the CT abnormalities within this small group did not allow us to evaluate biomarker concentrations in relation to different types of ICI. Finally, another limitation of the study was the lack of treatment information, which would have helped to determine the ability of UCH-L1 and GFAP to predict the need for intervention, such as neurosurgical procedures. This will be an important avenue for future investigation.

In conclusion, the results of the current exploratory study point to a role of GFAP and UCH-L1 as candidate biomarkers for pediatric TBI. As a traumatic insult on a developing brain may affect future brain maturation, neural connectivity, and children’s ultimate functional capabilities^46^, serum biomarkers might complement clinical and imaging data improving diagnostic accuracy and serve as objective evidence of TBI even in the presence of a normal head CT. Serum biomarkers could also potentially help guide patient selection for the use of advanced neuroimaging techniques. However, our data indicate that only UCH-L1 may function as a reliable biomarker for the detection of acute intracranial lesions as assessed by CT in pediatric TBI. Furthermore, GFAP and UCH-L1 might be used to identify children at risk of poor outcome thereby providing theranostic use both in clinical care and research. Further validation studies with larger numbers of patients will be required to assess the reproducibility of these findings and to confirm the potential clinical utilities of these markers in the setting of pediatric TBI.

## Materials and Methods

### Subjects

This prospective cohort study included a convenience sample of children suffering from TBI admitted to the Emergency Department at Children’s Hospital of Pittsburgh of UPMC (CHP). The protocol was approved by the University of Pittsburgh Institutional Review Board. Informed consent was obtained from parents. The study was conducted in accordance with the principles of the Declaration of Helsinki and the institutional guidelines.

Children were eligible for enrollment as cases if they were less than 15 years of age, with a history of an isolated closed head injury, had a head CT as part of clinical care and had blood collected for biomarker measurement within 24 hours of injury.

Children were eligible as controls if they were less than 15 years of age and had blood collected as part of an evaluation in which there was no concern for closed head injury (e.g. viral illness, routine bloodwork during well-child care).

### Measures

#### At time of presentation

All children received standard care for their injuries. A GCS score was assigned on arrival by the trauma team for each patient, as is standard practice in our institution. Demographic and clinical characteristics including age, gender, GCS and the result of the initial head CT were collected. Injury severity was classified as mild (GCS score 13–15), moderate (GCS score 9–12) or severe (GCS score ≤ 8). CT scans were evaluated as part of routine clinical care by an experienced pediatric neuroradiologist who was blinded to biomarker information. In subjects who had more than one CT scan performed, data were collected for the first CT. For the purposes of analysis, CT scans were classified as ‘positive’ if there was evidence of intracranial injury, either hemorrhage, contusion or edema, ‘skull fracture only’ if there was an isolated skull fracture without ICI and ‘negative’ if there was no skull fracture or ICI.

#### Biomarker measurement

Blood was collected as soon as possible after arrival to the hospital. Blood samples were centrifuged at 5,000 RPM for 10 minutes. Serum was removed, aliquoted in single vials to ensure a single freeze/thaw cycle per aliquot and then stored at −80^o^ C until analysis. Blinded sample analysis was conducted in a central laboratory (Banyan Biomarkers, LLC). Serum UCH-L1 was measured using a standard UCHL1 sandwich ELISA protocol as previously described^13^,^47^. Serum GFAP protein was analyzed employing electro-chemiluminescent immunoassay method (ECL-IA) using the MSD platform (MesoScale Discovery, Gaithersburg, MD)^27^. Serum S100B and MBP concentrations had been measured as part of previously published studies by our group^21^,^22^.

#### Outcome assessment

Outcome was assessed according to the Glasgow Outcome Scale (GOS)^48^ by the clinical team at hospital discharge and/or by a trained research technician at a scheduled follow-up clinic visit. The clinician performing the GOS was unaware of the serum biomarker concentrations. For statistical analysis, the GOS obtained at 6 months post-injury was considered and was dichotomized into favorable (GOS 4–5) or unfavorable (GOS < 4).

### Statistical Analysis

Statistical analyses were carried out using Stata software (Version 13.0, Stata Corp LP, USA) and SAS (version 9.0; SAS Institute Inc., Cary, NC). Data were assessed for equality of variance and distribution. Descriptive statistics with means and median, as appropriate, and proportions were used to describe continuous and categorical variables. For statistical comparison of biomarkers values, non-normally distributed continuous variables, we used the Mann–Whitney U test in case of two groups and the Kruskal- Wallis test in case of three or more groups. To test significant trend for increasing biomarker concentration in a given injury severity group (e.g. mild vs. moderate vs. severe) we used the Jonckheere-Terpstra test for non-parametric trend analysis. Correlation analyses were performed by means of the nonparametric Spearman rank correlation test. Spearman partial correlation analysis was used to adjust for the effect of other potential confounding variables. ROC curve analysis was used to calculate diagnostic accuracy of brain damage biomarkers for distinguishing between children with TBI and controls, for detecting ICI and for predicting a poor outcome. The area under the ROC curve (AUC) indicates the discriminative ability, ranging from 0.5 (random guessing) and 1 (perfect discrimination). As, in controls, serum UCH-L1 and S100B were associated with age, this covariate was incorporated into the analysis and age-adjusted ROC curves were estimated where appropriate. All statistical tests were two-tailed and a *p* value < 0.05 was considered significant.

## Additional Information

**How to cite this article**: Mondello, S. *et al*. Serum Concentrations of Ubiquitin C-Terminal Hydrolase-L1 and Glial Fibrillary Acidic Protein after Pediatric Traumatic Brain Injury. *Sci. Rep.* **6**, 28203; doi: 10.1038/srep28203 (2016).

## Figures and Tables

**Table 1:** Summary of demographic and clinical characteristics of the study population.

	TBI – All (n = 45)	GCS 3–8 (n = 19)	GCS 9–12 (n = 6)	GCS 13–15 (n = 20)
Age, years, (mean ± SD)	3.8 ± 3.7	2.8 ± 3.7	6.2 ± 4.5	4.1 ± 3.7
Gender, (M/F), n (%)	28/17 (62/28)	9/10 (47/52)	5/1 (83/17)	14/6 (70/30)
Race, n (%)				
White	31 (69)	14 (74)	4 (67)	13 (65)
Non-White	14 (31)	5 (26)	2 (33)	7 (35)
Lesion Types on Head CT, n (%)^a^				
Epidural hemorrhage	4 (0.8)	2 (11)	1 (17)	1 (5)
Subdural hemorrhage	17 (38)	10 (53)	1 (17)	6 (30)
Subarachnoid hemorrhage	5 (11)	2 (11)	–	3 (15)
Intracerebral hemorrhage	5 (11)	5 (26)	–	–
Intraventricular hemorrhage	4 (9)	4 (21)	–	–
Contusion	1 (2)	1 (5)	–	–
Edema	8 (18)	6 (32)	–	2 (10)
Skull Fracture	19 (42)	6 (32)	5 (83)	8 (40)
GOS 6 months, n (%)^b^				
Unfavorable (GOS 1–3)	11 (27.5)	11 (65)	–	–
Favorable (GOS 4–5)	29 (72.5)	6 (35)	4 (100)	19 (100)

GCS = Glasgow Coma Scale score; GOS = Glasgow Outcome Scale.

^a^Some patients had a combination of lesions.

^b^Missing outcome in 5 patients.

**Table 2:** Serum concentration of GFAP, UCH-L1, S100B and MBP.

	Cases (n = 45)	Controls (n = 40)	P value^b^
GFAP (ng/ml)	0.48 (0.12–1.67)	0.01 (0.00–0.05)	<0.0001
UCH-L1 (ng/ml)	0.23 (0.12–0.55)	0.09 (0.03–0.11)	<0.0001
S100B (ng/ml)	0.03 (0.02–0.06)	0.02 (0.015–0.023)	<0.0001
MBP (ng/ml)	0.15 (0.03–0.21)	0.17 (0.08–0.21)	0.39

^a^Data are given as median (interquartile range).

^b^Mann-Whitney U test.

**Table 3:** Serum GFAP and UCH-L1 concentrations in cases stratified by neuroimaging results.

		N	GFAP (ng/mL)	UCH-L1 (ng/mL)
Cases	Positive CT (ICI)	29	0.73 (0.15–2.28)+++	0.44 (0.17–0.99)+++
	Skull Fracture Only	6	0.58 (0.09–1.59)++	0.14 (0.08–0.22)+*
	Negative CT	10	0.21 (0.08–1.37)+++	0.14 (0.08–0.27)+**

ICI = intracranial injury. Symbols indicate statistical difference of the Mann-Whitney test for differences between groups: vs Controls (^+++^) p < 0.001, (^++^) p < 0.01, (^+^) p < 0.05; vs Positive CT (*) p < 0.05, (**) p < 0.01.
